# Shining light on motivation, emotion, and memory processes

**DOI:** 10.3389/fnbeh.2015.00001

**Published:** 2015-01-20

**Authors:** Anton Ilango, Mary K. Lobo

**Affiliations:** ^1^Department of Systems Physiology of Learning, Leibniz Institute for NeurobiologyMagdeburg, Germany; ^2^Department of Anatomy and Neurobiology, University of Maryland School of MedicineBaltimore, MD, USA

**Keywords:** emotion, reward, motivation, addiction, habits, obesity, optogenetics

Over the last 10 years, methods that combine genetics and optics have neuroscientists glowing about the possibilities to study movement, motivation, reinforcement, emotion and abnormal reward seeking processes such as addiction and obesity. From the fruit fly, rodents, to primates, optogenetics sheds light on the previously unanswered questions. With its specificity and high temporal precision, we gained several insights about the brain processes and we were the witnesses of those changes in the field.

Search with the key word “Optogenetic” retrieved close to 1500 articles in PubMed, almost more than 90% of them published in the past 5 years with tremendous progress in several areas of Neuroscience research. This includes dissection of neural circuitries combined with a vast array of behavioral procedures while simultaneously obtaining neurotransmitter measurement and electrophysiological signatures. These developments led us to deconstruct a detailed view of circuits underlying neuropsychiatric disorders. From studying reward, aversion, anxiety, associative learning, habit formation, the tools also applied to study the long lasting and persistent memories, which are bad for the healthy brain (for example, fear and addiction). The scope of the present research topic “*Neural circuits underlying emotion and motivation: Insights from optogenetics and pharmacogenetics*” is to provide a panoramic view of recent progress in this direction, with special emphasis on circuit level exploration of emotion, motivation and memory. A number of topics are covered which are of great interest to a broad readership. These include reinforcement, motivation under the influence of drugs compulsive consumption of food reward, and formation of habits and reactivation memory. Some of the reviews explicitly addressed the problems and the necessity of developing the next generation tools. We believe, leveraging the knowledge obtained from this research topic in Behavioral Neuroscience will help us to gain deeper insights in the future.

## From eliciting reinforcement to addictive processes

Dopamine (DA) has been implicated in functions ranging from motor control, motivated behavior, reward, associative learning, and mood regulation. Considering the enormous number of articles and the recent surge in exploring the dopamine system utilizing optogenetics, it is important to provide technical and critical analyses of behavioral function using different light stimulation parameters. In this research topic, two such studies appeared. Bass et al. (2013) demonstrated that distinct patterns of DA cell activation were able to disrupt the alcohol-drinking behavior of rats. Specifically applying the precise pattern of photo stimulation, resulting in low but prolonged levels of DA release (tonic), could prevent the rats from alcohol drinking. Ilango et al. (2014) studied role of phasic DA stimulation in supporting reinforcement by addressing the effect of various stimulation parameters on induction of intracranial self-stimulation. Subsequently, effects of interval and ratio schedules of reinforcement on phasic photo stimulation reinforcement were studied, which was also contrasted with food reinforcement. They found that phasic DA signal briefly enhance the initiation of approach behavior without long-term motivational regulation.

Activation of ventral tegmental area (VTA) DA neurons elicits reward and GABA induces aversion. Creed et al. (2014) discussed about the role of VTA GABA neurons to elicit aversion and their role in modulating associative learning based on their local control on DA neurons and striatal cholinergic interneurons. Another review article by, Cachope and Cheer (2014) discuss about the local control (i.e., the endogenous glutamatergic and cholinergic activity) of striatal DA release and relate their implications on potential mechanisms to modify impaired control of DA release in the diseased brain.

Environmental cues are powerful to reinstate a behavior after extinction. Stimuli previously associated with drugs of abuse can trigger drug-seeking behavior. Stefanik and Kalivas (2013) study describes the specific role of basolateral amygdala (BLA) projection either directly into the nucleus accumbens (NAc), or indirectly via the prelimbic prefrontal cortex (PL), on cue induced reinstatement of drug seeking. Also, long-lasting, drug-induced plasticity within the NAc have been proposed to contribute to drug-mediated addictive behaviors. Song et al. (2014) studied the role of NAc medium spiny neurons (MSNs) expressing dopamine D2 receptors (D2Rs) in cocaine-induced behavioral sensitization. The photo stimulation parameters did not affect the initiation nor the expression of cocaine-induced behavioral sensitization but attenuated the expression when applied during the withdrawal period.

## Pain, emotion, and memory

Pain elicits emotional responses and several studies suggests that these emotive events persist long term in the memory compared to events lacking an emotional component.

Carr and Zachariou (2014) reviewed studies using optogenetic stimulation of nociceptors in *C. elegans* and mice, which provided insights into the nociceptive circuitry and behavior, in both acute nociception and chronic pain states. In their conclusion, they stressed the importance of optopharmacology and novel optoreceptors in pain research with the added advantage of moving this into clinical research.

The amygdala has been linked to the regulation of emotion and affective behavior. Here, LaLumiere (2014) reviewed the recent discoveries on amygdala functioning on emotion, memory and addiction based on recent optogenetic experiments.

In another research article, Hübner et al. (2014) dissected the pathways implicated in fear and emotional memory *ex vivo*. They characterized cellular and synaptic responses of BLA neurons that where optogenetically activated by either medial prefrontal cortex (mPFC) or ventral hippocampus (vHC) inputs. Notably, the authors report that both mPFC and vHC inputs recruit feed forward inhibition in BLA, yet inputs from these structures differ in their presynaptic properties.

Ramirez et al. (2014) elegantly reviewed the concept of “memory engram” using fear conditioning experimental procedures. They narrated the history of the biological conceptualization of memory and then further to describe the biological locus and molecular signatures of memory. From their work, they described the process of identifying, activating and optogenetically incepting the false memories.

It is known that modulating serotonin (5-HT) levels in humans and animals affects perception and response to social threats, but there was little knowledge on the circuit mechanisms that control 5-HT dorsal raphe nucleus (DRN) output during social interaction. Here, Challis et al. (2014) studied the top-down control of 5-HT neurons by examining the vmPFC that project to the DRN in social approach-avoidance decisions.

## Food addiction and formation of habits

Krashes and Kravitz (2014) focus on reviewing the advances in understanding the feeding circuitry using optogenetics and pharmacogenetics (a.k.a. chemogenetics). After discussing the circuitry, which mediates homeostatic feeding, they sufficiently theorized and narrated the relevance between drug craving literature with compulsive consumption of food reward.

Our everyday life is influenced by habits but still the mechanism underlying the regulation of habits (the repetitive, chunking response patterns) is insufficiently understood. Here, Smith and Graybiel (2014), discuss multiple distinct components observed in the contrasting dynamics of neural activity from infralimbic cortex and dorsolateral striatum during different stages of habit learning.

## Need for the next generation tools and new procedures

In their opinion article, Kravitz and Bonci (2013) discuss about the state of the neural activity during optogenetic stimulation and discuss the firing parameters evoked by the stimulation outside of physiological ranges.

Sidor and McClung (2014) minireview article, focused on the diurnal specific control of neural activity and the necessity of chronic stimulation paradigms and technological advances.

Ting and Feng (2014) provided insights on the development and application of diverse BAC transgenic rodent lines and discuss about the under-appreciated issue of “extra” genes contained within the large BAC DNA sequences. In their research article, they propose simple and reproducible strategies for developing the next generation of BAC transgenic lines that are devoid of extra genes which are very much helpful across several disciplines.

Despite the common practice of photo stimulating the circuitry to activate the opsins, the study by Land et al. (2014) reported a new method using the internal generated light source based on firefly luciferase. They co-infected striatal neurons with halorhodopsin and luciferase-expressing viruses in the striatum followed by treatment with luciferin. Through reduced Fos activation, reduced neuronal firing, and blunted amphetamine induced locomotion; they demonstrated a feasible method to optogenetically inhibit neuronal activity through luciferase, without the need for optic fiber implants.

Given the thorough exploration and insights accomplished in this Research Topic, we hope that you will enjoy these new stages of exciting intersections between the neurobiology of emotion, motivation and memory. We appreciate the reviewers' insights during the review process and we greatly acknowledge their effort. It was for us a great pleasure to collect, read, and edit these manuscripts. We hope, you will profoundly enjoy reading this research topic as well.

### Conflict of interest statement

The authors declare that the research was conducted in the absence of any commercial or financial relationships that could be construed as a potential conflict of interest.

## References

[B1] Bass C. E. Grinevich V. P. Gioia D. Day-Brown J. D. Bonin K. D. Stuber G. D.. (2013). Optogenetic stimulation of VTA dopamine neurons reveals that tonic but not phasic patterns of dopamine transmission reduce ethanol self-administration. Front. Behav. Neurosci. 7:173. 10.3389/fnbeh.2013.00173 24324415 PMC3840465

[B2] Cachope R. Cheer F. (2014). Local control of striatal dopamine release. Front. Behav. Neurosci. 8:188. 10.3389/fnbeh.2014.00188 24904339 PMC4033078

[B3] Carr F. B. Zachariou V. (2014). Nociception and pain: lessons from optogenetics. Front. Behav. Neurosci. 8:69. 10.3389/fnbeh.2014.00069 24723861 PMC3971183

[B4] Challis C. Beck S. G. Berton O. (2014). Optogenetic modulation of descending prefrontocortical inputs to the dorsal raphe bidirectionally bias socioaffective choices after social defeat. Front. Behav. Neurosci. 8:43. 10.3389/fnbeh.2014.00043 24596546 PMC3925846

[B5] Creed M. C. Ntamati N. R. Tan K. R. (2014). VTA GABA neurons modulate specific learning behaviors through the control of dopamine and cholinergic systems. Front. Behav. Neurosci. 8:8. 10.3389/fnbeh.2014.00008 24478655 PMC3897868

[B6] Hübner C. Bosch D. Gall A. Lüthi A. Ehrlich I. (2014). *Ex vivo* dissection of optogenetically activated mPFC and hippocampal inputs to neurons in the basolateral amygdala: implications for fear and emotional memory. Front. Behav. Neurosci. 8:64. 10.3389/fnbeh.2014.00064 24634648 PMC3943336

[B7] Ilango A. Kesner A. J. Broker C. J. Wang D. V. Ikemoto S. (2014). Phasic excitation of ventral tegmental dopamine neurons potentiates the initiation of conditioned approach behavior: parametric and reinforcement-schedule analyses. Front. Behav. Neurosci. 8:155. 10.3389/fnbeh.2014.00155 24834037 PMC4018564

[B8] Krashes M. J. Kravitz A. V. (2014). Optogenetic and chemogenetic insights into the food addiction hypothesis. Front. Behav. Neurosci. 8:57. 10.3389/fnbeh.2014.00057 24616674 PMC3937547

[B9] Kravitz A. V. Bonci A. (2013). Optogenetics, physiology, and emotions. Front. Behav. Neurosci. 7:169. 10.3389/fnbeh.2013.00169 24312032 PMC3833017

[B10] LaLumiere R. T. (2014). Optogenetic dissection of amygdala functioning. Front. Behav. Neurosci. 8:107. 10.3389/fnbeh.2014.00107 24723867 PMC3972463

[B11] Land B. B. Brayton C. E. Furman K. E. LaPalombara Z. DiLeone R. J. (2014). Optogenetic inhibition of neurons by internal light production. Front. Behav. Neurosci. 8:108. 10.3389/fnbeh.2014.00108 24744708 PMC3978322

[B12] Ramirez S. Tonegawa S. Liu X. (2014). Identification and optogenetic manipulation of memory engrams in the hippocampus. Front. Behav. Neurosci. 7:226. 10.3389/fnbeh.2013.00226 24478647 PMC3894458

[B13] Sidor M. M. McClung C. A. (2014). Timing matters: using optogenetics to chronically manipulate neural circuitry and rhythms. Front. Behav. Neurosci. 8:41. 10.3389/fnbeh.2014.00041 24592222 PMC3924037

[B14] Smith K. S. Graybiel A. M. (2014). Investigating habits: strategies, technologies, and models. Front. Behav. Neurosci. 8:39. 10.3389/fnbeh.2014.00039 24574988 PMC3921576

[B15] Song S. S. Kang B. J. Wen L. Lee H. J. Sim H. Kim T. H.. (2014). Optogenetics reveals a role for accumbal medium spiny neurons expressing dopamine D2 receptors in cocaine-induced behavioral sensitization. Front. Behav. Neurosci. 8:336. 10.3389/fnbeh.2014.00336 25352792 PMC4195370

[B16] Stefanik M. T. Kalivas P. W. (2013). Optogenetic dissection of basolateral amygdala projections during cue-induced reinstatement of cocaine seeking. Front. Behav. Neurosci. 7:213. 10.3389/fnbeh.2013.00213 24399945 PMC3871970

[B17] Ting J. T. Feng G. (2014). Recombineering strategies for developing next generation BAC transgenic tools for optogenetics and beyond. Front. Behav. Neurosci. 8:111. 10.3389/fnbeh.2014.00111 24772073 PMC3982106

